# IPv6 Addressing Proxy: Mapping Native Addressing from Legacy Technologies and Devices to the Internet of Things (IPv6)

**DOI:** 10.3390/s130506687

**Published:** 2013-05-17

**Authors:** Antonio J. Jara, Pedro Moreno-Sanchez, Antonio F. Skarmeta, Socrates Varakliotis, Peter Kirstein

**Affiliations:** 1 Computer Science Faculty, University of Murcia, Murcia 30100, Spain; E-Mails: p.morenosanchez@um.es (P.M.-S.); skarmeta@um.es (A.F.S.); 2 Department of Computer Science, University College London, London, WC1E 6BT, UK; E-Mails: s.varakliotis@cs.ucl.ac.uk (S.V.); p.kirstein@cs.ucl.ac.uk (P.K.)

**Keywords:** internet of things, 6LoWPAN, legacy technologies, IPv6, addressing, naming

## Abstract

Sensors utilize a large number of heterogeneous technologies for a varied set of application environments. The sheer number of devices involved requires that this Internet be the *Future Internet*, with a core network based on IPv6 and a higher scalability in order to be able to address all the devices, sensors and things located around us. This capability to connect through IPv6 devices, sensors and things is what is defining the so-called *Internet of Things* (IoT). IPv6 provides addressing space to reach this ubiquitous set of sensors, but legacy technologies, such as X10, European Installation Bus (EIB), Controller Area Network (CAN) and radio frequency ID (RFID) from the industrial, home automation and logistic application areas, do not support the IPv6 protocol. For that reason, a technique must be devised to map the sensor and identification technologies to IPv6, thus allowing homogeneous access via IPv6 features in the context of the IoT. This paper proposes a mapping between the native addressing of each technology and an IPv6 address following a set of rules that are discussed and proposed in this work. Specifically, the paper presents a technology-dependent IPv6 addressing proxy, which maps each device to the different subnetworks built under the IPv6 prefix addresses provided by the internet service provider for each home, building or user. The IPv6 addressing proxy offers a common addressing environment based on IPv6 for all the devices, regardless of the device technology. Thereby, this offers a scalable and homogeneous solution to interact with devices that do not support IPv6 addressing. The IPv6 addressing proxy has been implemented in a multi-protocol card and evaluated successfully its performance, scalability and interoperability through a protocol built over IPv6.

## 1. Introduction

The Future Internet and IPv6 protocol present a new generation of capabilities and access to the network, which will extend the Internet seamlessly to personal devices, smart phones and sensors, making a reality of what is called the *Internet of Things* (IoT) from a purist communications point of view [1].

Current sensors and their application environments consist of a vast group of technologies that lack efficient interoperability among them [2]. Some associations of manufactures have been formed to build a common technological framework in specific application domains, e.g., Konnex (KNX) [3] for building automation, ZigBee with an independent alliance (ZigBee Alliance) [4], and other protocols, such as X10 and Controller Area Network (CAN), which are totally independent. These protocols are not interoperable. For that reason, many designers of the Internet of Things are considering a move toward a common access and communication framework based on IPv6. This change to the internet protocol will affect the addressing of the devices for each legacy technology; a common addressing at the device level is mandatory. Otherwise, one would require the use of portal servers or gateways in order to reach real machine-to-machine (M2M) communications.

This paper presents a mechanism for the users and devices to map the different addressing spaces to a common IPv6 one. Thereby, information and communication technology convergence can be reached through the Internet homogeneity. Specifically, every device from each technology will operate through a common framework based on IPv6 and protocols over IPv6, such as web services, e.g., RESTful and Constrained Application Protocol (CoAP) [5,6] or any other protocol via TCP/UDP sockets.

In the different application areas, many different technologies for sensors and ubiquitous computing are available. A migration strategy, as much as possible, should be capable of integration into the IoT [7]. The resolution of this problem in view of the large number of such technologies is the motivation for this paper. Most of the technologies were designed in a context of small and local networks, with limited capabilities; the requirements and design issues were quite different from the current situation. For example, several years ago, when X10 was defined, only a small number of sensors would be placed in the same building or a single control area. The same applied for European Installation Bus (EIB)/KNX, which was one of the entrance technologies to offer an extended deployment of sensors in home and building automation. Nowadays, sensors are found almost everywhere. Indeed, their number will increase exponentially in a short period of time with the definition of the smart grid, smart cities and the demand to connect every device to the Internet for full connectivity and interoperability via technologies, such as ZigBee-IP [4,8,9], 6LoWPAN [10,11] and GLoWBAL IPv6 [12].

The IoT with technologies, such as ZigBee-IP, 6LoWPAN, GLoWBAL IPv6 and the extension of legacy technologies to IPv6, will allow the management of everything around us and the acquisition of information automatically and independently of the technology used. However, this presents a challenge, since each technology has its own idiosyncratic features. For this reason, this paper proposes and discusses how specific differences among the wide spectrum of technologies can be hidden through the IP abstraction.

For this purpose, it is necessary to define an IPv6 mapping for the native addressing of the devices. These mapping techniques will be presented for X10, EIB/KNX, CAN and radio frequency ID (RFID). For each technology, technology-specific features will be mapped to a set of fields defined within the IPv6 address. Thereby, this will allow the location and identification of the devices in a multi-protocol card either as presented in [13] from our previous works or in any gateway or portal server, such as [14].

In summary, this work presents the idea of an IPv6 addressing proxy and how it can do the translation between an IPv6 address and its corresponding technology address, depending on the technology in the Section 2. This mapping requires information for each technology. For that reason, the following sections give an overview about every technology used for this IPv6 mapping in the Section 3. Finally, evaluation and results are presented in Sections 5 and 6.

## 2. IPv6 Addressing Proxy

While multiple devices and sensors will be connected to the Internet, each group of sensors may use its own technology. Each device needs to send data frames through the Internet to other devices and systems connected remotely. This makes it desirable that each sensor has an IP address; this will have to be an IPv6 one, due to the high number of existing and future sensors.

Up to now, each device, which takes part in an IoT network, had an arbitrary IPv6 address to reach the Internet. In this paper, two mechanisms for address provision are considered: one offers support for an auto-configuration process requiring a mapping table; the other uses a non-arbitrary address. Our work is focused on this second option, since it avoids the definition and maintenance of mapping tables, making it more scalable.

The IPv6 address is formed by the *network ID* and *host ID*; the device identification is located at the *host ID* part. Therefore, this paper defines how to configure and divide the *host ID* into different IPv6 fields. Thereby, the **IPv6 addressing proxy** will be able to transform the native addressing and the *line device ID* to the *IPv6 host ID* and *vice versa* in a way that is dependent on *technology*.

The mapping has been mainly focused on the 64 bits available for the *host ID* in an IPv6 address. However, since the networks can present a netmask lower than */64*, for example, */48* or */56*, these bits available in the network ID to build subnetworks can be also used for the purpose of the identification of the described *technology* and *line ID* fields. This consideration to also build subnetworks taking into account the *technology* or *line ID* field is to allow that the technology discrimination can be carried out by the networking modules (routing). Thereby, part of the pre-processing can be relayed to the kernel. Additionally, this relay to the kernel also allows reusing other IP-related tools, such as *iptable*, for managing the access control policies for the different technologies and line IDs, since they will be represented by different networks.

**Figure 1.**
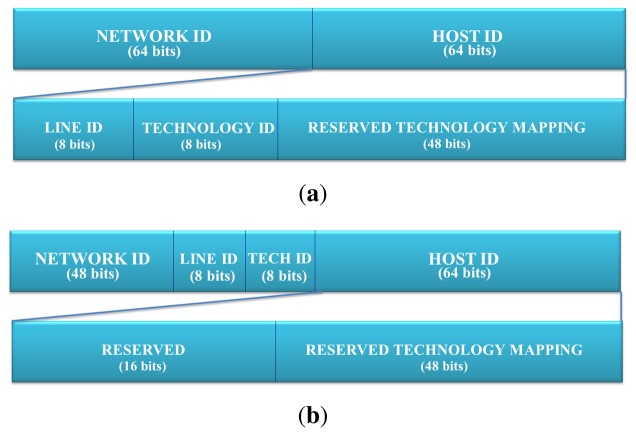
IPv6 address format in the IPv6 addressing proxy. (**a**) Addressing functionality in application level; (**b**) Addressing functionality within kernel.

Specifically, there are two ways proposed to implement the technological discrimination in the function of the prefix length offered by the internet service providers (ISP). Figure 1 presents both schemes. On the one hand, proxy addressing can be done at the application level, due to the fact that we have a unique component of the network address for every technology, *i.e.*, a netmask of 64 bits (see Figure 1(a)). On the other hand, in the scheme outlined in Figure 1(b), an IPv6 network for each couple, {technology id, line id}, is necessary. When it is available, a netmask should be under 64 bits. Specifically, an ISP should provide an IPv6 network of 48 bits of netmask for each home, building and user; most of the mechanisms used in IPv6 assume that it will have 48 bits of netmask in the network address [15]. This will allow the technology and line identifier to be contained within the *network ID* and to define a subnetwork for each technology.

The fields from the Figure 1 have the following meaning:

- **Line ID.** (8 bits) This field contains an identifier of the line to which the sender device belongs. It can differentiate up to 255 lines. It is useful in some technologies, like X10, where multiple transceivers co-exist, or in the case of EIB/KNX, where the network is split into areas and zones. Therefore, the *line ID* is used to discriminate a subnet governed by a specific transceiver. Finally, this has been defined as a security and management mechanism in order to differentiate groups of the same technology.
- **Technology ID.** (8 bits) This field contains the identifier of the technology used by the sender device. This allows the **IPv6 addressing proxy** to determine the technology used for matching with the corresponding data contained in the *reserved technology mapping*.
- **Reserved Technology Mapping.** (48 bits) This field is divided into multiple fields depending on the technology specification. The following subsections present the mapping for different technologies of this field.

**Figure 2.**
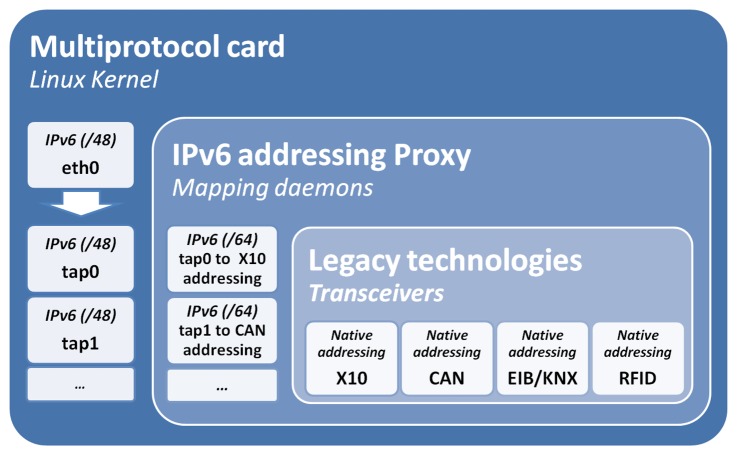
IPv6 addressing proxy functionality and mapping hierarchy.

For the rest of this work, it will be considered that there are 48 bits of netmask, although the argument can be adapted to a 64 bit netmask. Moreover, in this example, we consider a situation in which there is only one legacy network of each technology connected to the multiprotocol card. This avoids the need of sub-netting the technology component. Figure 2 describes the mapping hierarchy and how this is managed by a set of virtual network kernel devices built with *tun*/*tap* interfaces.

*Tun*/*tap* interfaces simulate a link layer device, which operates with layer 2 packets, such as Ethernet frames. Specifically, tap interfaces are used as bridges (simulate MAC level—layer 2), and tun are used for routing (simulate network level—layer 3). This work uses this technology to build virtual interfaces to distinguish between the different technologies. This presents a different usage to the original one for tunneling, bridging or routing.

The main motivation to build a virtual interface is to make sure that the IPv6 addressing proxy is listening to the IPv6 mapped addressing spaces. In addition, the kernel provides several advantages from IP packet management, such as routing (*route*) and security (*iptable*).

Mainly, *tap* interfaces are in this proposal to bridge between the transceivers driver and the Ethernet interface (eth0). Thereby, packets received at the Ethernet interface (*eth0*) are listened to by a set of daemons added to the operating system. For each technology, its transceiver is linked to a software driver implemented in the user space. Similarly, the *tap* interfaces, *i.e.*, the transceivers and daemons can also deliver packets to the Ethernet interface in order to reach a full duplex solution.

## 3. IPv6 Mapping for Legacy Technologies

The mapping has been carried out initially for a representative home automation technology, *i.e.*, X10, a building automation technology, *i.e.*, EIB/KNX, a industrial technology, *i.e.*, CAN, and, finally, a logistics and identification technology, *i.e.*, RFID.

### 3.1. X10

**Figure 3.**
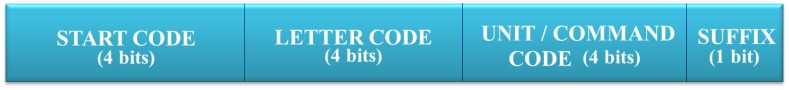
X10 data frame.

X10 is a standard that defines a codification format to be transmitted via Power Line Carrier (PLC) [16]. X10 is organized around four basic principles: asynchrony, locality, atomicity and order. Generally, a X10 data frame is composed of bits distributed as presented in the Figure 3. The meaning of these fields is:

- **Start Code.** (4 bits) Always set to 1110.
- **Letter Code.** (4 bits) House address code. It is used by the receiver node to identify the house where the sender node is located.
- **Unit/Command Code.** (4 bits) Unit address/command code. This field represents the unit address in the first half of the data frame. In the second half of the data frame, this field is used for sending the command identifier.
- **Suffix,** (1 bit) If this bit is set to 0, the previous field indicates the unit code. However, if the suffix field is set to 1, the previous field is the code of the command to be executed.

**Figure 4.**
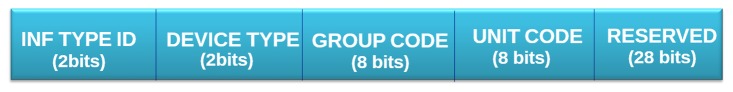
IPv6 mapping from X10 address.

X10 device addressing only needs two fields, *letter code* and *unit code*. Therefore, it only requires 8 bits to make a X10 device address. However, in this case, we have extended the number of bits for mapping to IPv6. Our proposal is presented in Figure 4.

The fields used in the presented mapping are:

- **Information Type Identifier.** (2 bits) This field is an identifier of the information carried. It can discriminate up to four different information types.
- **Device Type.** (2 bits) X10 installations are composed of different types of devices. In our installation, there will be sensors, actuators and transceivers.
- **Group Code.** (8 bits) This field matches the *letter code* field in the X10 data frame. However, it is composed of 8 bits to support extended networks.
- **Unit Code.** (8 bits) This field matches the *Unit Code* field in the X10 data frame (4 bits). However, it is composed of 8 bits in order to extend the possibilities in the device numbering to group tasks. At the moment, the value, 0×00, for the additional 4 bits is reserved to indicate that the message is a group query and every device must receive the message.
- **Reserved.** (28 bits) This field must be set to 0 by default. This field will be used in the future when new features are designed, such as extra information about the devices and security, e.g., digital certificates and signatures, as in ORCHID identifiers [17].

### 3.2. EIB/KNX

The European Installation Bus (EIB) [3] is an open and comprehensive system that covers all aspects of building automation. It has been designed as a management system in the field of electrical installations for load switching, environmental control and security for different types of buildings. EIB can be used for a lot of functions, according to [18].

EIB/KNX defines different frame formats, depending on the communication medium and service. The most commonly encountered format by far is the twisted-pair (TP) standard data frame, which we have considered to carry out the presented mapping.

Focusing on the addressing in EIB/KNX for TP [18], it is necessary to understand the EIB/KNX topology. Basically, the individual addressing starts in lines. The lines are grouped by areas, and finally, we can have up to 15 areas connected by a main line, called a backbone. In addition to individual addressing, EIB/KNX supports logical/group addressing, which is used to associate a group of devices with similar functionality.

To the network stack, the logical/group address is an entirely opaque identifier that bears no relation to either the content of the message or the receivers' individual addresses. Integrators assign group addresses based on the information provided or the function to be effected by the associated group objects (e.g., site-wide functions/panic alarm). No binding standard interpretation of the group-address space is specified by the KNX standard. For this reason, the logical/group address mappings are optional, but are maintained, since they can be used in the half-gateways and mapping application layer to identify the devices to their legacy EIB/KNX network.

The proposed mapping takes into account these addressing characteristics of both individual and logical/group addressing, in order to make this mapping easier.

**Figure 5.**
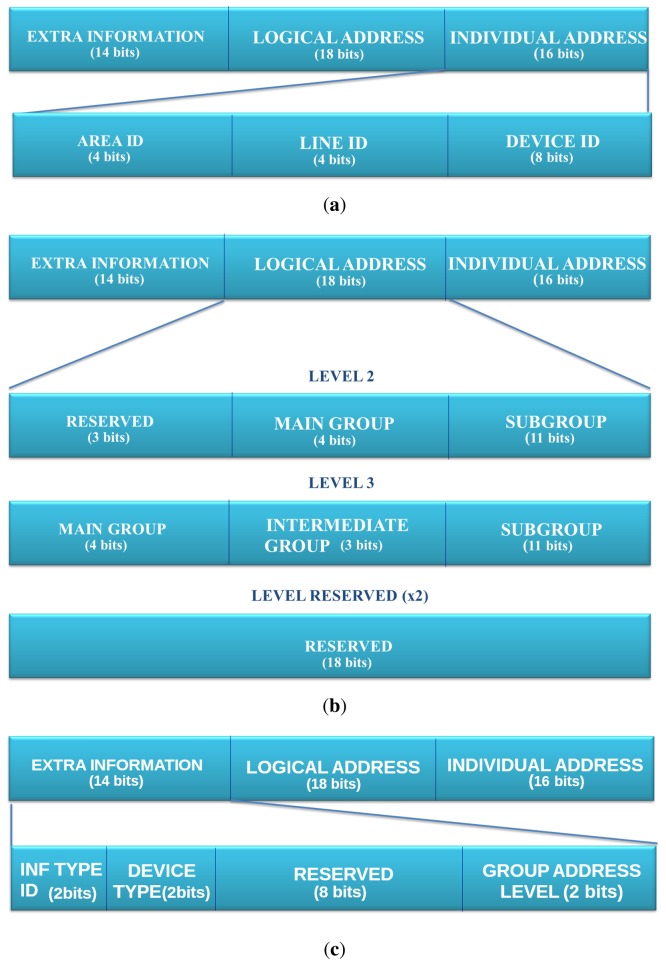
IPv6 mapping from European Installation Bus (EIB)/Konnex (KNX). (**a**) Individual address mapping; (**b**) Logical/group address mapping; (**c**) Extra bits mapping.

Figure 5(a) presents how the individual address structure is maintained in the less significant bits (16 bits). These fields contain the information about the *device identifier* and *the line and area identifiers* to which the sender device belongs. Secondly, Figure 5(b) presents an overview of the mapping with the individual address. There are two extra individual address formats that can be used to set up the correct field in the *extra information* part of the IPv6 address. Finally, Figure 5(c) presents how the rest of the bits are distributed. These 14 bits have been spread in four fields as follows:

- **Information Type ID.** (2 bits) An identifier of the information's type that the device is able to manage. It can discriminate four different types of information.
- **Device Type.** (2 bits) EIB installations can use unto four different types of devices; this field discriminates the type of device. The matching between the value of this field and the device type are sensor, actuator, line coupler and area coupler.
- **Group Address Level.** (2 bits) This field discriminates the logical level addressing, which is being used.
- **Reserved.** (8 bits) This field can be used to map the priority field, although this brings the problem that the a single device will allocate multiple IPv6 address or any other usage required by the application.

**Figure 6.**
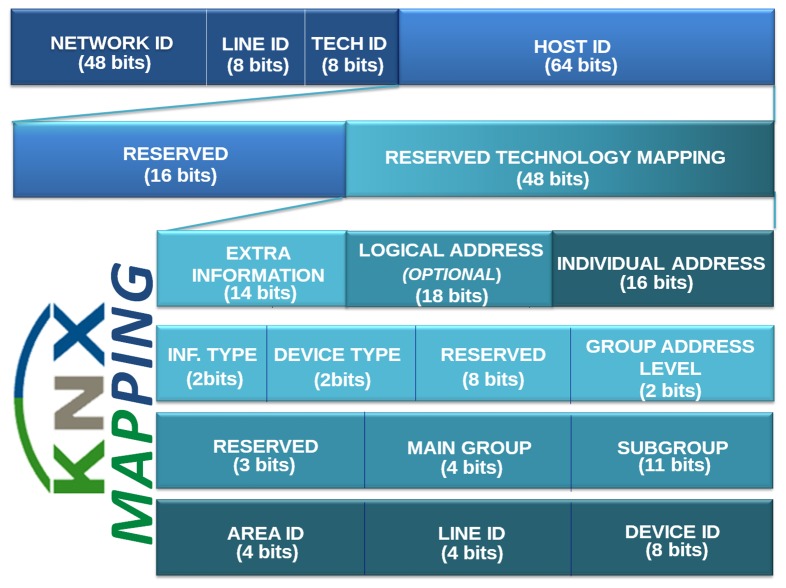
Full mapping from host ID to the EIB/KNX address.

An example of full mapping for a device EIB/KNX to the IPv6 address is found in Figure 6. This presents from the *host ID*, upon which is mapped extra information, logical address and individual address.

### 3.3. CAN

The Controller Area Network (CAN) [19] is a serial communications protocol that supports efficiently distributed real-time control with a very high level of security [20]. The CAN application domain covers high speed networks to low cost multiplex wiring in automotive electronics, engine control units, sensors, anti-skid-systems, *etc.*

**Figure 7.**
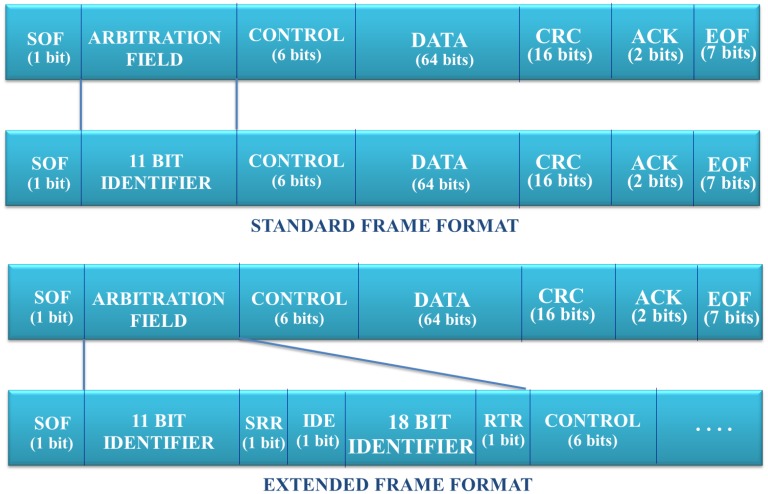
CAN bus data frame.

Figure 7 presents the bit distribution in a CAN bus data frame. From all the fields of a CAN data frame, this mapping is focused mainly on the *arbitration field*, since this contains the addressing/location information. This data field length depends on the type of the data frame used in the message exchange. There are two data formats available in CAN:

- **Standard Frame Format.** This format defines 11 bits to identify the sender device.
- **Extended Frame Format.** This format defines a bigger number of bits in order to identify the devices. Concretely, this uses 29 bits for this purpose.

**Figure 8.**
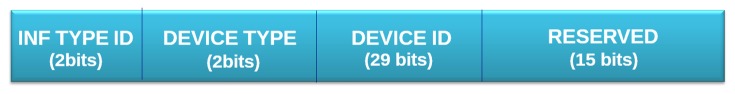
IPv6 mapping from CAN.

This mapping considers the identifier defined by the *extended frame format*, which also supports the *standard frame format*. In addition, it is proposed to use 19 bits more in order to support a bigger range of features. Figure 8 presents the fields:

- **Information Type Identifier.** (2 bits) This is an identifier of the information carried in the message. The system administrator is in charge of determining the matching between each value and its corresponding information type.
- **Device Type.** (2 bits) This field is used to identify the device type used in the CAN communication, *i.e.*, sender, receiver or controller.
- **Device Identifier.** (29 bits) This field contains the identifier of the device. This field matches the original *identifier* field present in the *extended frame format* of the CAN standard. In the case of using the *standard frame format*, the 11 least significant bits must be used for identifying the device, and the rest of bits of this field must be set to 0. In this way, we have support of the two types of identifier size existing.
- **Reserved.** (15 bits) This field must set to 0 by default. This field might be used for some new features according to a system's administrator plan. For example, this field can be used for identifying the site in which the device is situated. With this information, we can apply security policies. For example, that a device cannot send a message to a device if this is not located in the same site. In addition, we can set the site identifier depending on its critical level. A critical site might be set to *000 0000*. A non-critical site should be set to *111 1111*. Notice that in the CAN standard, the lower values have more priority than the bigger ones.

### 3.4. RFID

RFID offers an extended set of standards, technologies and flexibility for the identification of its smart labels. The most deployed protocol and identifier is the Electronic Product Code (EPC) from EPCGlobal [21]. EPC codes are 96-bit unique codes. They are used for short-distance (high frequency—HF) solutions, such as ICODE EPC SL2 ICS 10, and for long-distance (ultra high frequency—UHF) with tags compatible with the ISO/IEC 18000-6 EPC CLASS1 GEN2 standard, such as U-CODE G2 XM. EPCGlobal proposes an architecture similar to that of the Internet for the management of the EPC; it consists of EPC information systems and a global object name server (ONS), which can be seen as the equivalent to the DNS.

A mapping between EPC and IPv6 is needed in order to integrate EPC over IPv6. This integration is justified, since EPC is not a unique standard for product identification. For the mapping between EPC and IPv6, a proposal can already be found, such as [22]. For that reason, we are not proposing any new mapping from EPC to IPv6.

In addition to EPC, a Unique Identifier (UID) can be used [23], which consists of a 40-bit identifier hard-coded by the manufacturer to ensure it is really unique. This uniqueness property is being considered by the pharmaceutical industry, because it satisfies the requirements to offer an efficient, trustable and safely traceable solution. In addition, it is able to verify the authenticity of a drug. UID is found in solutions, such as ICODE UID SL2 ICS 11 and MiFare. The integration of UID in IPv6 also makes the homogeneity of the IoT through the use of IPv6 feasible.

**Figure 9.**
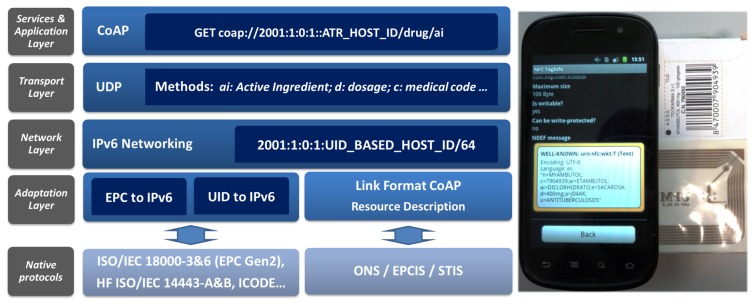
Radio frequency ID (RFID) integration into IPv6 and the Internet of Things; example of access to a tag with the description of the services in Constrained Application Protocol (CoAP) link format.

Figure 9 presents an example of the application of an identifier of drugs from a drugs checker to prevent adverse drug reactions [24]. In addition, since UID-based tags offer additional space to store information, further attributes are defined, which are accessible through web services, such as CoAP and RESTful.

This reads the tag, which contains extended information in link format, such as for CoAP; this information is accessible through the IPv6 address by mapping the 40 bits from the UID into the host ID of the IPv6 address, in a way similar to the one presented for EIB/KNX. The information contained in the tag, e.g., an active ingredient description, is subsequently transferred to the RFID reader, making it globally accessible through the Internet.

The RFID implementation has a prototype in a smart phone; since the mapping is carried out via the RFID/NFC interface, any communication performance has not been determined. The next section will present the evaluation and implementation for the other presented use cases, *i.e.*, X10, KNX and CAN, over a multi-protocol board.

## 4. Functionality Bridging between Legacy Technologies and the Internet of Things

The Internet of Things attempts to link any kind of device, the so-called everything, to the Internet. Thereby, it can be accessed in a homogeneous and interoperable way.

This is part of the efforts carried out by the information and communication technology (ICT) community to reach an ICT convergence. IP has been considered a major enabler in building a common connection framework, where all the devices are reachable among them. Therefore, the definition of mechanisms to map legacy addressing spaces to IPv6 is required, and for that purpose, IPv6 addressing proxy has been presented.

The solution is not only to locate devices through IPv6 address; the mapping and adaptation towards common protocols and semantic descriptions is also required.

The Internet of Things is considering web services as the candidate to define the application framework. Specifically, CoAP [6] is a lightweight version of HTTP and RESTful architecture for this kind of solution.

For this purpose, the IoT6 European Project [25] has defined an application-level proxy that abstracts away from the underlying legacy network, e.g., map functions from legacy technologies, such as X10 and EIB/KNX.

**Figure 10.**
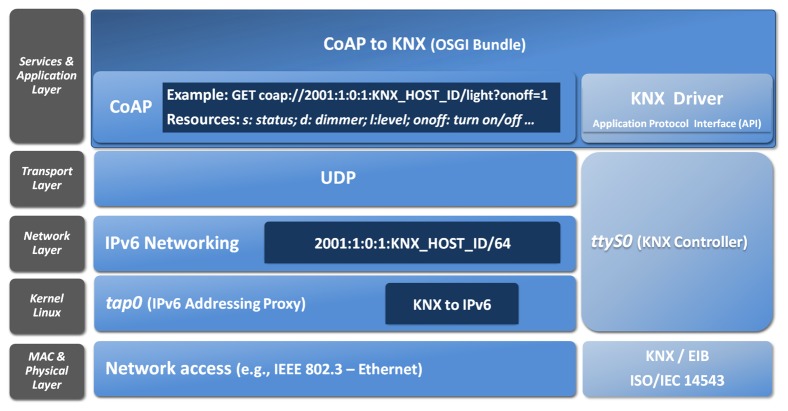
Communication stack for bridging between legacy technologies and the Internet of Things.

Figure 10 presents the mapping and bridging carry out for EIB/KNX. Thereby, an IPv6 addressable EIB device can be also have its functionality accessed through a set of CoAP-based methods.

The semantic in that case is being built with Open Building Information Exchange (oBIX) [26], since this defines a neutral standard interface based on RESTful web services for building automation systems. Although this originally used XML for the exchange of data, it has been optimized to support JSON in the context of the IoT6 EU project in [27].

Specifically, the mentioned bundle to map from CoAP to KNX is defined in the IoTSyS [28]. IoTSyS is an integration middleware for the Internet of Things, which provides the application-gateway for existing sensor and actuator systems found in home and building automation systems nowadays.

In addition to the mentioned CoAP/oBIX approach, another approach from the IoT6 EU project to demonstrate the integration of legacy devices into IPv6 is *Turn It IPv6* [29]. Turn It IPv6 enables communication with legacy devices using their proprietary protocols, such as Zigbee sensors, KNX valves and X10 actuators. At the same time, it offers an IPv6 addressing and application proxy between IPv6 and those devices, each device having its own public IPv6 address, such as offered by the IPv6 addressing proxy. The application-proxy is based on the universal device gateway technology [30] developed by Mandat International (IoT6 EU project coordinator).

## 5. Evaluation

### 5.1. Testbed Overview and Implementation Issues

**Figure 11.**
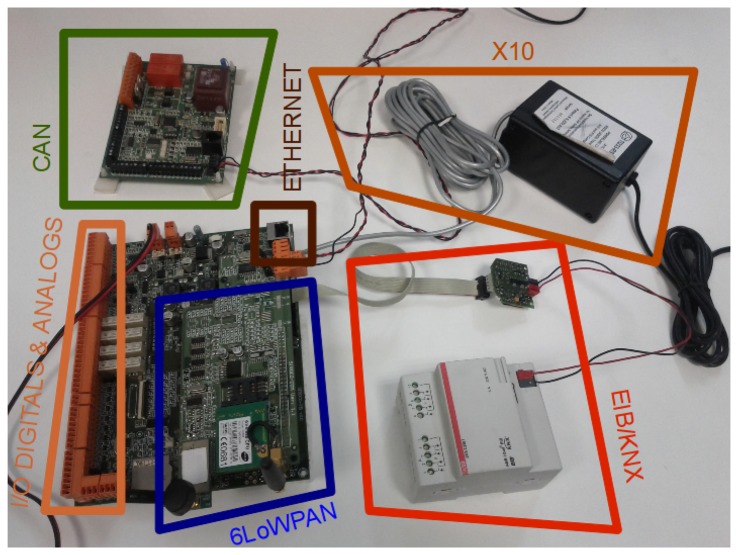
Multi-protocol card used for the evaluation.

The **IPv6 addressing proxy** has been validated in a multi-protocol card from previous work [13], which is presented in Figure 11.

This multi-protocol card is based on the Atmel ARM9 processor running at 400 MHz (32 bit) with 256 MB LPDDR RAM and 256 MB NAND memory, which supports the Linux OS. This board supports, through its extension interfaces (serial RS232 and SPI ports), the technologies for industrial and building automation. More specifically, the extension interfaces support Control Area Networks (CANs), X10 and European Industrial Bus (EIB)/Konnex (KNX).

**Figure 12.**
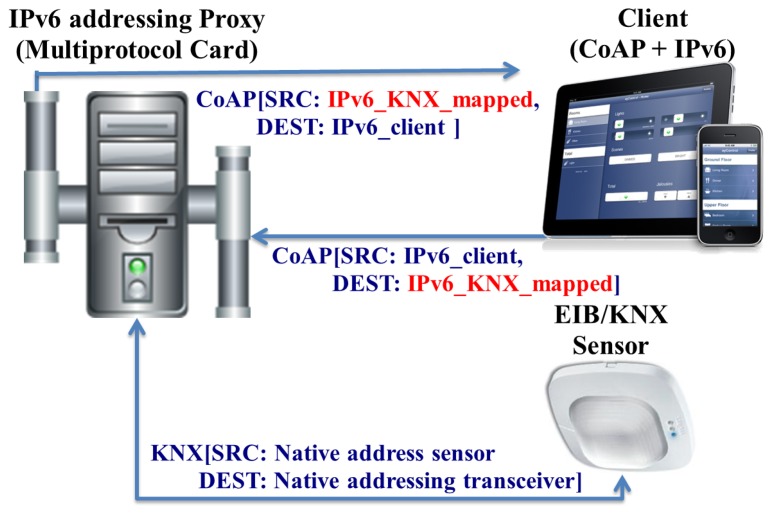
Evaluation framework.

This multi-protocol board is in charge of mapping between the multiple technologies and their corresponding IPv6 addresses and *vice versa*. That functionality is divided in the following modules (see Figure 12 with the deployment scheme).

The evaluation scenario is composed of the following software modules and clients:

- **Transceiver Driver—**Software in the multi-protocol card manages the traffic generated from the legacy technologies to the core network. This software identifies and has detailed knowledge about the node's technology; this is needed to build the IPv6 address following the mapping techniques presented earlier. The software listens to the native interfaces in accordance with the technology used for each device (e.g., X10, EIB/KNX, CAN or RFID), *i.e.*, serial ports.
- **Receiver Module (Daemon)—**Software in the multi-protocol card manages the incoming traffic from the core network to the specific sensor system. This software listens for messages sent by the *clients*. Once a message is received, this receiver decodes the IPv6 destination address and technology. It follows the mapping specifications in order to make a message in the format of the requisite technology.
- **Remote Client Application—A** client in this infrastructure is some device that receives information about a sensor location, e.g., a remote server. This client has two options. On the one hand, it can be aware of the mapping and try to understand the native features of the device from a transformation of the IPv6 address information related to the technology being used. On the other hand, a recommended option is to abstract data from the technology and to define homogeneous web services based on RESTful/CoAP.

### 5.2. Mapping Processing

We will focus on an explanation of the mapping processing in the *receiver module (daemon)*.

First, this defines the function, *check technology*; its operation will depend on where the *technology id* is allocated. Second, after the *receiver* knows with which technology it should be used, work in the mapping processing is then carried out in the parsing. The following subsections present the mapping processing for both phases.

#### 5.2.1. Technology Pre-processing

As described in Section 2, the integration of the mapped address is part of the host ID in the IPv6 address. Additionally, the mapping of the technology and line ID in the network ID can be considered in order to exploit the routing functionality provided by the networking kernel in order to manage the first pre-processing mapping focused on technology and area (line ID) classification.

This second option, where part of the pre-processing is carried out at the networking layer, depends on the network prefix provided by the ISP. Particularly, ISPs are considering to provide /56 and /48 bits for residential and commercial buildings, respectively.

For that reason, considering /56 bits, 255 different subnetworks based on technology can be defined. In the same way, with /48 bit, we have the opportunity to make a difference between 255 different areas and 255 different technologies per area.

Finally, in the case that the ISP provides a single network, *i.e.*, /64 bit, such as for end-clients or cellular networks, network routing cannot be exploited to make a difference between technologies and areas, and consequently, this pre-processing needs to be carried out by the IPv6 addressing proxy daemon.

#### 5.2.2. Legacy Address Parsing

Once the *receiver* knows which technology should be used in the mapping process, it is necessary to take care of some typical situations in the IPv6 parsing:

- A field is set to 8 bits, corresponding to a complete byte. This case is the easiest, and we only have to check the value of this byte.
- A field is set to a number of bits smaller than 8 within a byte. In order to extract it, we need a division; we will obtain the value if it is at the beginning, or we will need to do a module operation if it is at the end of the byte.
- A field is set in different bytes. In this case, we will need to obtain the value of each one and to do a multiplication for the corresponding power of 2 in each byte.
- A field is set in two different bytes, so this needs to extract the corresponding part of each byte and reset the value of each part in their proper proportion.

The most complex case is the mapping specification. In order to decrease the delay and increase the scalability of the mapping processing, multiple daemons can be defined, each listening to a different interface, rather than having only one daemon listening to every request on one network interface. For that reason, we also considered the possibility of discriminating the technology at the network layer instead of the application one.

The best case for this routing-based discrimination would be if the ISP provides us with an IPv6 network with 48 bits of netmask. In this case, we would be able to set up different networks, and we could set up a virtual interface for each network. In this scenario, the kernel is in charge of managing the routing of the message to the correct interface.

To take full advantage of this scheme, in our case, we had to set up three daemons—one for each technology. This is because a daemon can listen only to one subnetwork. Indeed, we might even need to define multiple daemons to one technology, carrying out a division per line, in order to increase the scalability. Here, we note that in the future, it will be possible to process the daemons in a parallel architecture (multiple CPUs).

In the case of only one daemon for all the technologies, it will need to check which technology is being used in the mapping. This is because, potentially, there can be requests from different technologies. The following subsection presents a performance evaluation comparing the two approaches.

For that reason, this evaluation is focused on, on the one hand, the evaluation of the overload and processing time required for the IPv6 addressing proxy through the mean time for the translation from each independent receiver module for each technology and, on the other hand, an evaluation about the scalability through the performance with a flood of requests in order to evaluate which of the approaches is more relevant, a common daemon or a daemon for each subnetwork/interface.

### 5.3. Evaluation Scenario

The scenario used to get the results shown in the article is depicted in Figure 12.

Both the sender and the receiver are run over the multiprotocol card, in which a specific operating system based on Linux distributions was used. The distribution used is Linux4Sam [31].

The client part was run over a laptop with Ubuntu 10.04 32 bits stripped as the operating system. Finally, the communication between both entities (multiprotocol card and laptop) was performed using an Ethernet connection.

The multiprotocol card and the laptop used in this evaluation process are running an operating system based on Linux. This fact simplifies the set up, due to the fact that the same configuration steps can be performed in both systems.

### 5.4. Evaluation Methodology

Two different modes of working have been tested. These modes differ in the number of daemon running at the same time in the receiver entity: first, a single daemon for all the technologies, and second a version with a daemon per technology (which requires three daemons).

This difference relies mainly on how the technology pre-processing phase is performed. When a single daemon is being performed, the client has to check the technology used in the host ID part of the IPv6 address. However, when a daemon per technology is deployed, then the technology pre-processing is directly carried out by the kernel using the network ID.

#### 5.4.1. A Single Daemon in the Receiver

**Figure 13.**
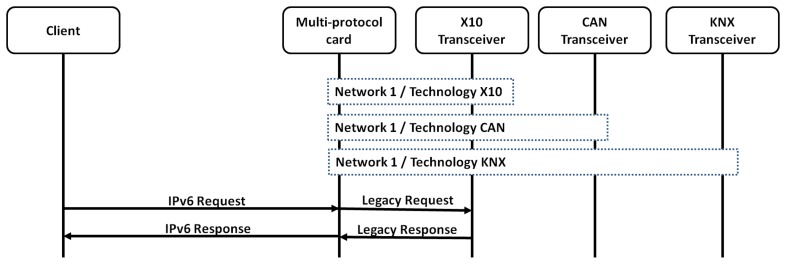
Evaluation Environment with a Single Daemon and Interface.

The scenario performed for a single daemon in the receiver is depicted in Figure 13. This presents as all the technologies share the same interface. For that reason, the daemon justs listen to all the IPv6 addressed defined for each one of the sensors, independently of the technology, over the same interface.

This requires an extra pre-processing, since this needs to analyze the technology and the parsing, while with a daemon per technology, the first pre-processing step is elided. For that reason, a solution with a daemon per technology is also evaluated.

#### 5.4.2. A Daemon Per Technology in the Receiver

**Figure 14.**
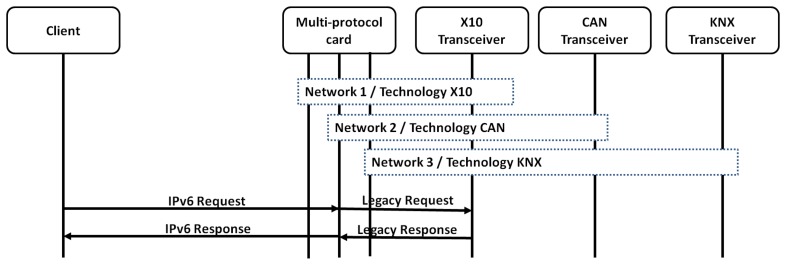
Evaluation environment with a daemon and interface per technology.

In this use case, the scenario used is that depicted in Figure 14. This has built a virtual interface (network) per technology.

In this use case, the advantage of using three daemons to listen to each technology, instead of a single daemon listening to all of the interfaces, has been evaluated. This advantage is only possible if each daemon is listening to incoming messages in a different interface.

Given that the multi-protocol card has only an Ethernet interface, the use of virtual interfaces is mandatory. For this purpose, tap/tun interfaces are used.

It has been needed to add tun/tap support to the kernel of the multi-protocol card. For that purpose, the *openVPN* [32] packet has been added. Once those features have been successfully added, the virtual interfaces can be created, such as presented in the following listing.


openvpn ––mktun ––dev tun–x10ip link set tun–x10 upip −6 addr add 2001:6 b0 : e : 0 0 0 0 ∷ 1 / 4 8 dev tun–x10openvpn ––mktun ––dev tun–canip link set tun–can upip ‒6 addr add 2001:6 b0 : e : 0 0 0 1 ∷ 1 / 4 8 dev tun–canopenvpn ––mktun ––dev tun–eibip link set tun–eib upip ‒6 addr add 2001:6 b0 : e : 0 0 0 2 ∷ 1 / 4 8 dev tun–eib

The configuration is a bit more complicated than in the single daemon use case, since additional networks to connect with each one of the daemons need to be configured as the receivers.

The tests have been based on performing the whole scenario multiple times to get several times the execution. Concretely, the scenario has been executed 900-times for each technology. For this purpose, a shell script has been used in order to run the sender in a loop. The client and the receivers are executed and listening to incoming requests once they are correctly handled. Finally, requests related to the three technologies have been performed.

The next section presents the results obtained.

## 6. Results

The results are performed in terms of processing time and scalability, such as described in the following subsections.

### 6.1. Processing Time Evaluation

For determining the processing time for the mapping process itself the mean time for the IPv6 Addressing Proxy mapping process has been evaluated.

The following charts present the time spent on the evaluation of the time for transmission from a sender to a receiver, plus the processing times on the sender and receiver sides.

The processing time depends on the mapping from IPv6 to legacy addressing and *vice versa*. For that reason, it is evaluated with and without mapping for each one of the different technologies for a set of 575 messages.

**Figure 15.**
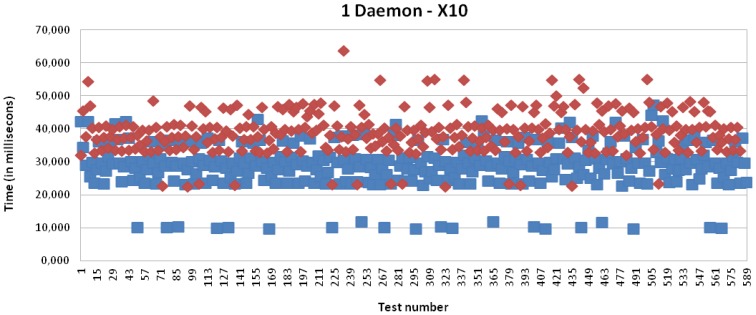
Transmission and processing time for X10 technology with IPv6 Addressing Proxy, *i.e.*, mapping (red) and without mapping (blue), when a single daemon is managing all the requests.

Specifically, Figure 15 presents the results for X10 technology with a single daemon; it is presenting an average of 39,042 milliseconds for the full process, where 22,876 milliseconds depends on the processing time in the multi-protocol card. The processing time in the case that the mapping from the IPv6 address to the legacy address is not carried out is equal to 13,571 milliseconds, *i.e.*, the time required to receive the packet from the kernel, process it and, finally, make it available to the application.

**Figure 16.**
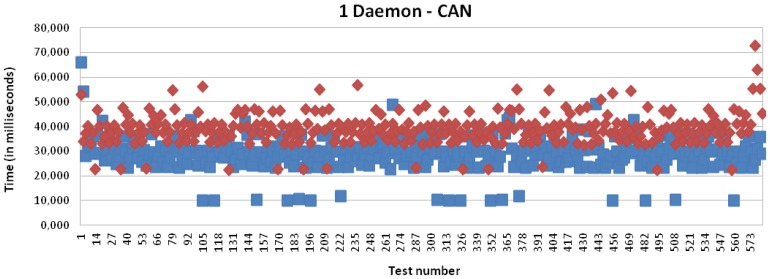
Transmission and processing time for CAN technology with mapping (red) and without mapping (blue), when a single daemon is managing all the requests.

**Figure 17.**
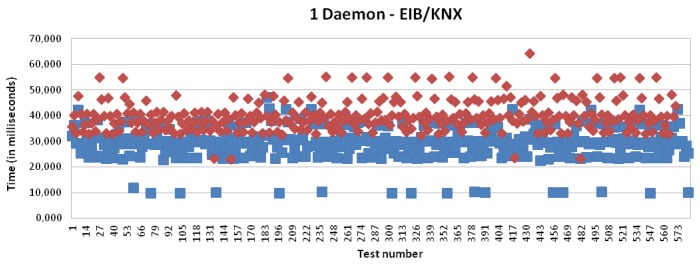
Transmission and processing time for EIB/KNX technology with mapping (red) and without mapping (blue), when a single daemon is managing all the requests.

Figures 16 and 17 present the results for CAN and EIB/KNX technology, respectively. They present similar results, such as described in more detail in Table 1.

It can be initially concluded analyzing these charts and summary table that the delay introduced by the IPv6 addressing proxy in the mapping process is negligible for the client side, which is based on an Intel Core i5 CPU. However, the delay introduced by the mapping process by the receiver side, *i.e.*, by the multi-protocol card, which is based on an Atmel ARM9 processor running at 400 MHz (32-bit), presents a mean of 10 milliseconds. In numbers, this presents a mean time of 9,997 milliseconds for X10, 10,550 milliseconds for CAN and 10,503 milliseconds for EIB/KNX. The little differences are mainly due to the involved mathematical operations, which introduce some complexity into the mapping operation, such as mentioned in the evaluation regarding the mapping processing tasks.

### 6.2. Scalability Evaluation

For the scalability evaluation of the IPv6 addressing proxy, multiple requests from a remote client have been carried out. Specifically, 1,725 consecutive requests have been run (575 for each technology). The times shown are averages from the 1,725 requests in a window time of one minute in order to evaluate the scalability of the buffers.

For this purpose, a daemon per technology (three daemons) has been defined in order to reduce the processing time in the receiver side, *i.e.*, the multi-protocol card.

**Figure 18.**
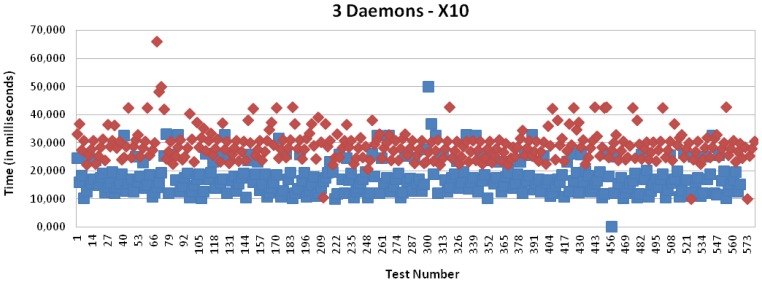
Transmission and processing time for X10 technology with mapping (red) and without mapping (blue), when three daemons are managing the requests.

**Figure 19.**
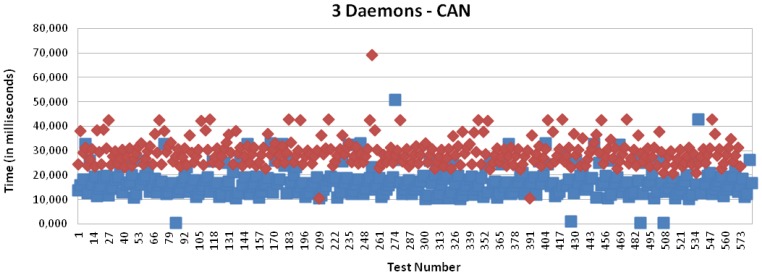
Transmission and processing time for CAN technology with mapping (red) and without mapping (blue), when three daemons are managing the requests.

**Figure 20.**
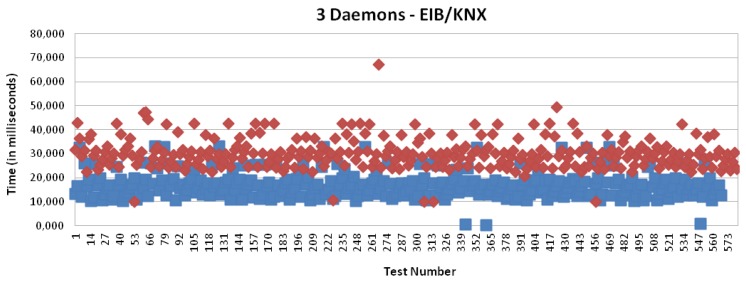
Transmission and processing time for EIB/KNX technology with mapping (red) and without mapping (blue), when three daemons are managing the requests.

Figures 15, 18, 19 and 20 present the results for X10, CAN and EIB/KNX technologies, respectively, when three daemons are being executed on the receiver side, each one managing a different technology.

**Figure 21.**
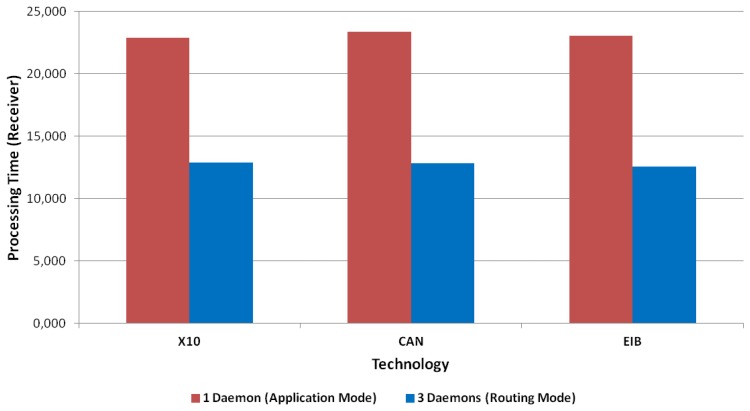
Average of times in the IPv6 mapping processing with application or routing modes.

Table 2 presents a summary of the results for three daemons with the different technologies. Finally, Figure 21 presents a comparison between the solution based on application (one daemon) and routing mode (three daemons). This shows a considerable reduction of the time. Clearly, the routing mode from the Linux kernel offers a more scalable solution than the management at the user level, because it allows multiple processing of packets with independent interruptions. In addition, this reduces the requirements for local buffers for queues. In the future, there could be further reduction by using platforms with multiple-cores for parallel processing.

The daemon per technology allows us to exploit the capabilities of the Linux kernel to pre-process the incoming packets to make the distinction among the different technologies through the routing capabilities, since each technology is allocated in a different subnetwork.

Another reason to present a higher delay in the application-mode with respect to the rerouting mode is that the routing made is carried out in kernel mode, while the application-mode requires a transition to the user mode in the Linux Operation System.

User mode presents a lower priority, and it is interrupted by any third party fault (e.g., page fault), interrupt (e.g., input/output), trap (e.g., a system call), *etc.*

## 7. Discussion

The proposed *IPv6 addressing proxy* has been implemented and evaluated in a multi-protocol card. This has required the provision of a daemon listening to all the connection in order to process the requests for any technology. Since this can present scalability problems, we have proposed and investigated experimentally a model of multiple daemons (usually one per technology), in order to make this solution more efficient and scalable. In our example, there was a protocol mapping time of around 8 ms. With only one daemon, the total processing time of a transaction was around 30 milliseconds; with three daemons, this was reduced to 12 milliseconds. Therefore, the delay introduced by the IPv6 addressing proxy is insignificant in practice. The approach is indeed a suitable mechanism to introduce into the IoT, in order to incorporate legacy technologies into a homogeneous, IPv6-based Internet environment.

Focusing on the mapping processing, this is not an essential element to consider, due to the absence of significant differences. However, the mapping processing time in the receiver needs to be considered, because there is a big gap between doing it and avoiding it. Concretely, there is an extra delay of 22 milliseconds if a single daemon is being used and a delay of 12 milliseconds when a daemon per technology is used.

The difference between these delay values corresponds with the two modes we can use. As theoretically supposed, using a daemon listening to each technology (three daemons in our work) is better than using only one daemon managing every technology mapping. This assertion is based on the data we have obtained in our performance tests and its evaluation.

The performance improvement is significant, according to the averages of the total time needed in one transaction; this improves this time with the one daemon per technology mode in every case tested: without mapping the improvement, it is 11 milliseconds. If the mapping process is tested, the improvement decreases, until 10 milliseconds, but also, it is a proof of the advantages of using a daemon per technology against a single daemon.

## 8. Conclusions

This work has considered the need to make legacy technologies accessible via the Internet to an end-to-end addressing mechanism based on IPv6. Thereby, end devices can be identified via the IPv6 address, and emerging protocols, such as CoAP, could be accessed by the native functionality. The proposed addressing proxies carry out this double task: on the one hand, the addressing mapping and, on the other hand, the functionality mapping from web services, such as RESTful, and the already mentioned CoAP to native protocols.

For this purpose, this work presents a set of mapping techniques between legacy technologies from home automation, industrial and logistic areas to IPv6. The mapping offers a homogeneous addressing and identification framework for the IoT.

The advantages from mapping legacy addressing spaces to IPv6 are:

- Mapping to IPv6 allows the hiding of the specific features of the technologies used by the sensor, in order to provide a framework that works independent of technology.
- A homogeneous environment to access the sensors based on IP technologies, such as web services.
- Anycast and multicast properties can be used to locate the IPv6 addressing proxy, since the transformation server can be separated from the transceiver drivers (*i.e.*, sensor controllers).
- Fault tolerance can be defined through multicast groups, removing single points of failure.
- Security can be defined with the use of different subnetworks for different technologies and groups inside of a technology via sub-netting. Thereby, access control policies based on the current IP-based solutions, such as *iptable*, can be applied.
- IPv6 addressing proxy enables the capability to manage traffic from legacy technologies in a homogeneous, flexible and standardized way via well-known IP-based protocols and mechanisms.

## Figures and Tables

**Table 1.** One daemon result for transmission and processing time.

**Time (ms)**	**Mapping**	**Average**			**Minimum**			**Maximum**		
		
		**X10**	**CAN**	**EIB**	**X10**	**CAN**	**EIB**	**X10**	**CAN**	**EIB**
Transmission	No	15,084	15,045	15,410	0,261	0,343	0,206	33,503	53,499	32,959
	Yes	16,166	15,828	16,975	0,088	0,239	0,450	41,653	39,806	32,996

Processing (client)	No	0,033	0,032	0,033	0,020	0,024	0,020	0,105	0,124	0,126
	Yes	0,033	0,033	0,033	0,020	0,022	0,020	0,110	0,107	0,107

Processing (receiver)	No	13,571	13,714	13,460	9,419	9,412	9,408	34,649	24,063	24,114
	Yes	22,876	23,354	23,055	20,828	20,865	20,870	34,170	45,159	44,778

Total	No	28,656	28,760	28,870	9,720	9,811	9,666	47,160	65,906	46,995
	Yes	39,042	39,182	40,030	22,444	22,464	23,028	63,821	72,872	64,167

**Table 2.** Three daemon results for transmission and processing time.

**Time (ms)**	**Mapping**	**Average**			**Minimum**			**Maximum**		
		
		**X10**	**CAN**	**EIB**	**X10**	**CAN**	**EIB**	**X10**	**CAN**	**EIB**
Transmission	No	17,575	16,918	16,747	0,254	0,263	0,269	50,023	50,803	32,892
	Yes	16,370	16,575	17,543	0,199	0,578	0,198	32,984	57,085	54,897

Processing (client)	No	0,031	0,034	0,033	0,018	0,018	0,020	0,050	0,121	0,052
	Yes	0,033	0,033	0,034	0,021	0,021	0,022	0,113	0,120	0,153

Processing (receiver)	No	0,110	0,226	0,112	0,043	0,044	0,044	11,295	22,790	12,112
	Yes	12,879	12,804	12,552	9,764	9,771	9,764	33,438	23,354	34,489

Total	No	17,685	17,144	16,859	0,328	0,340	0,344	50,070	50,879	32,400
	Yes	29,249	29,127	30,095	66,038	10,395	10,007	66,038	69,297	67,178
